# On Modern Progress in Ophthalmic Medicine and Surgery

**Published:** 1895-10-05

**Authors:** Robert Brudenell Carter

**Affiliations:** Consulting Ophthalmic Surgeon to St. George's Hospital.


### On Modern Progress in Ophthalmic Medicine and Surgery.

By Robert Brudenell Carter, F.R.C.S., Consulting Ophthalmic Surgeon to St. George's Hospital.

### SQUINT.

There are few departments of ophthalmic surgery in which so much progress has been made, within the memory of persons still living, as in the treatment of squint. The definition of squint is a condition in which the two eyes are not directed to the same point; and it is obvious that the deviation may be of one or both, and that it may be of any direction for either. In technical language, squint was for many years only called "strabismus," a modern derivative of the Latin "strabo," with the addition of convergent, or divergent, to express the ordinary directions of deviation. Sursumvergent and deorsumvergent are met with in a few writings, but the cases requiring these appellations are comparatively rare, and the more familiar "upward" and "downward" are quite sufficient to describe them. Lately, however, a new nomenclature has been introduced, and squint is now often called "heterophoria"; while corresponding barbarisms, such as "exophoria," "esophoria," and the like, have been coined to indicate the direction.

A pronounced squint is a powerful factor in the production of ugliness; and, perhaps on this account, has often been popularly associated with equally pronounced moral obliquity. In the novels of the last, or of the earlier portion of the present century, a squint was generally bestowed upon the villain of the story. In those days squint must have been very common, no operation for its relief having become established; and it was not until near the middle of this century that tenotomy of the over-acting rectus muscle can with any certainty be said to have been performed. A century earlier there flourished one John Taylor, M.D., who, in 1734, published a treatise on cataract, which he dedicated to Queen Caroline, mentioning, in his dedication, that he had attended Her Majesty in his professional capacity. He seems to have been somewhat peripatetic in his habits, for he mentions having practised in Paris, and puts after his name titles obtained from several foreign universities. In 1739 he published at Lisbon a book entitled, "De Vera Causa Strabismi," of which I have not been able to obtain a copy. Apparently, however, he must have recommended some form of operation, for, in 1752, Eschenbach, of Rostock, published an account of the effects of Taylor's operation, and describes him as "Ritter von Taylor," so that he had probably been knighted. Eschenbach's book is mentioned in Professor Alfred Græfe's catalogue of writings on squint, but it is not in the library of the College of Surgeons, and I have not been able to find it elsewhere, so that the nature and results of Taylor's operation are alike unknown to me. Judging from his book on cataract, Taylor had a large element of the charlatan in his compotion, and his operation for squint, if it had been successful, would scarcely have been suffered to fall into oblivion.

In 1838 Stromeyer, of Hanover, following Delpech in his experiments with regard to the treatment of the deformities occasioned by abnormal muscular contraction, suggested that squint might be regarded as one of the number, and that it might be cured by division of the rectus tendon on the side of the deviation. He even demonstrated upon the dead subject the manner in which division of the tendon might be effected, and Dr. Pauli, of Landau, soon afterwards attempted to operate upon a child seven years of age. He was completely baffled by the struggles of the little patient, and by the want of any contrivance for keeping the eyelids apart, and only succeeded in making a useless incision into the conjunctiva. In 1839, on October 26th, Dieffenbach operated successfully, on the lines laid down by Stromeyer, and published a little book on the subject, a second edition of which was called for in the following year. He gives a minute account of the steps of his procedure, and then bursts out into the following rhapsody:—

"I confess that the good result of this first operation for squint has afforded me the most lively satisfaction that I have experienced in the whole of my scientific career, since the importance of a means of removing one of the most objectionable of deformities must be obvious. The news of my success was spread over the whole civilised world with a rapidity unexampled in relation to science, and like that which attends the diffusion of political intelligence. The newspapers of Germany, France, and America, as well as of other countries, soon contained accounts of hundreds of similar operations; while, besides newspapers, my own pupils carried the knowledge over land and sea, and everywhere found imitators."

In his second edition, Dieffenbach says that he had already operated on 218 cases, and Baudens, writing on the same subject in 1841, in Paris, says that he has operated upon between eight and nine hundred. Such was the prevalence of squint before a remedy for it had been discovered.

Dr. Franz, presumably one of Dieffenbach's pupils, was the first to perform the operation in England, but I have not found any record of his result or any subsequent mention of his name. Mr. Bennett Lucas operated on April 7th, 1840, and soon became the recognised London authority upon the subject. He improved upon Dieffenbach's operation by rendering it more simple, and published a little book which went through three or four editions.

In the writings of that period I have found no records of failure, but it is certain that many must have occurred, and that the consequences of the operation must often have been exceedingly disappointing to those upon whom it was performed. Prior to the discovery, by Donders, that squint is produced by an error of refraction, a fault in the shape of the eyeball, it was universally attributed to a fault originating in the affected muscle, and the tenotomies performed for its relief were seldom graduated in such a manner as to produce the desired change of position and no more. Many of the operations of those days were excessive in degree; and the ordinary convergent squint was frequently changed into one still more disfiguring in an opposite direction. I once knew an unfortunate gentleman who was compelled to cover one eye with a shade for the rest of his life, in order to conceal the effect of an operation for convergent squint, which had been performed upon him in the early forties.

